# Pathogen distribution characteristics and risk factors of pulmonary infection associated with immune checkpoint inhibitor therapy in patients with advanced non-small cell lung cancer: a case control study

**DOI:** 10.1186/s12879-026-13834-1

**Published:** 2026-06-25

**Authors:** Ruixin Tian, Xiaoman Cui, Lantao Wang, Li Yan, Ping Wang

**Affiliations:** 1 Department of Respiratory Medicine, The Fourth Hospital of Hebei Medical University, Shijiazhuang, 050011 China; 2 Emergency Department, The Fourth Hospital of Hebei Medical University, Shijiazhuang, 050011 China; 3 Department of Respiratory and Critical Care Medicine, Hebei General Hospital, Shijiazhuang, 050011 China

**Keywords:** Non- small cell lung cancer, Immune checkpoint inhibitors, Pulmonary infection, Risk factors

## Abstract

**Background:**

To investigate the pathogen distribution and risk factors for pulmonary infection (PI) in patients with advanced non- small cell lung cancer (NSCLC) treated with chemotherapy combined with immune checkpoint inhibitors (ICIs).

**Methods:**

Patients with advanced NSCLC who met the inclusion criteria were collected and divided into three groups: chemotherapy with PI group, chemotherapy combined with ICIs with PI group and chemotherapy combined with ICIs without PI group. We displayed the pathogen distribution and infection time of PI for different groups, and explored the risk factors for PI in chemotherapy combined with ICIs group of patients.

**Results:**

A total of 239 patients were included in the study, among which there were 30 cases in the chemotherapy with PI group, 68 cases in the chemotherapy combined with ICIs with PI group, and 141 cases in the chemotherapy combined with ICIs without PI group. The results revealed an increased bacterial infection rate of patients in the chemotherapy with PI group (*p* = 0.003). Data analysis of patients with combined fungal infections showed a statistically significant difference in the incidence of fungal infections between the two groups (Fisher’s exact test, *p* = 0.035). Multivariate logistic regression analysis revealed that low lymphocyte (LYM) counts (*P* = 0.016) and stage IV disease (*P* = 0.012) were independent risk factors for patients in the chemotherapy combined with ICIs with PI group, and number of immunotherapy cycles ≥ 5 (*P* = 0.031) was associated with a reduced likelihood of PI.

**Conclusions:**

In this observational study, bacterial infections were more common with chemotherapy alone, whereas fungal infections predominated in patients receiving chemotherapy plus ICIs. Low lymphocyte count and stage IV disease were associated with higher pulmonary infection risk, while ≥ 5 cycles of immunotherapy was associated with lower risk.

## Background

As one of the most common malignant tumors in the world, the mortality rate of lung cancer is showing a slow downward trend, but the disease burden remains extremely heavy [1]. Relevant data show that the mortality rate of lung cancer is gradually decreasing, yet the number of deaths caused by lung cancer still exceeds the total number of deaths from breast cancer, prostate cancer, colorectal cancer and brain cancer, which is an issue that still deserves our attention [2]. Chemotherapy is the basic treatment for advanced non-small cell lung cancer (NSCLC). With the continuous development of anti-tumor drugs, immune checkpoint inhibitors (ICIs) have been widely used in the treatment of advanced lung cancer. Mainly consisting of PD-1/PD-L1 monoclonal antibodies, ICIs have become the first-line/second-line standard treatment regimen for advanced lung cancer, which significantly prolong the overall survival (OS) and progression-free survival (PFS) of patients. Some patients with advanced lung cancer have achieved long-term survival, greatly improving the treatment prognosis of patients with advanced NSCLC [3, 4]. At the same time, the incidence of pulmonary infections and immune-related pneumonitis (CIP) has increased significantly, which has become an important clinical problem that limits the benefits of immunotherapy, leads to treatment interruption and even death [5, 6]. ICIs enhance antitumour effects by relieving T- cell immunosuppression but may also disrupt the balance of the pulmonary immune microenvironment, increasing the risk of infection [7]. In addition, the use of combination chemotherapy, corticosteroids, or antibiotics may further suppress immune function, leading to pneumonia or fungal/bacterial infections [8].

When lung cancer is complicated by infection, its clinical management becomes more complex, and the prognosis of patients may significantly worsen. Therefore, exploring reliable biomarkers or diagnostic strategies (such as bronchoalveolar lavage and metagenomic sequencing) is crucial for optimizing clinical decisions. Studies have shown that the mortality rate of lung cancer patients complicated by infection is significantly greater than that of checkpoint inhibitor-related pneumonia (CIP) patients [9]. Therefore, studying the characteristics of pulmonary infections in patients with advanced non-small cell lung cancer (NSCLC), clarifying the high-risk factors for infections (such as advanced age, underlying pulmonary diseases, combined chemotherapy, long-term use of glucocorticoids, etc.), pathogen spectrum characteristics and onset rules, can provide a scientific basis for the clinical formulation of precise infection screening, prevention and hierarchical treatment plans. For example, based on the stratified high-risk groups identified by the research, closer pulmonary monitoring, preventive anti-infection measures and other interventions can be taken for NSCLC patients with interstitial lung disease and advanced age when using immunosuppressants to reduce the incidence of infections; aiming at the differences in pathogen spectrum found in the research, the selection of anti-infection drugs can be optimized to avoid the abuse of antibiotics, improve the effectiveness of treatment, and ultimately ensure the smooth implementation of immunosuppressant therapy, balancing the therapeutic efficacy and safety.

This study aims to explore the pathogen distribution characteristics and risk factors for concurrent pulmonary infection (PI) in advanced non-small cell lung cancer (NSCLC) patients treated with chemotherapy combined with ICIs. It also seeks to clarify the spectrum of pathogens complicating infections, provide a basis for precision treatment, and identify strategies for improving the outcome and quality of life of cancer patients.

## Methods

### Patients

In this study, patients diagnosed with advanced NSCLC who received treatment in the respiratory medicine ward of the Respiratory Department, the Fourth Hospital of Hebei Medical University between January 1, 2022, and December 31, 2023 were retrospectively included. Baseline clinical data were collected from medical records. Patients were followed up for survival status until September 1, 2025. Patients with advanced NSCLC who received chemotherapy combined with ICIs as first-line therapy, or those who received chemotherapy alone, were included in the study. Based on whether they had PI and the treatment they received (chemotherapy combined with ICIs or chemotherapy alone), the patients were divided into three groups: those who received chemotherapy combined with ICIs and had PI, those who received chemotherapy combined with ICIs but did not have PI, and those who received chemotherapy alone and had PI. The study was approved by the Ethics Review Committee of the Fourth Hospital of Hebei Medical University (ethics number 2023KS206).

### Data collection standards

Inclusion criteria: (1) age range of 18 to 80 years including boundary values; (2) initial histopathological diagnosis of NSCLC; (3) TNM stage from IIIB to IV; (4) negative gene testing results (including EGFR, ALK, ROS1, BRAF V600, KRAS, MET, RET, and NTRK); (5) first-line treatment with chemotherapy combined with ICIs or simple chemotherapy; (6) expected survival period ≥ 12 weeks. Exclusion criteria: (1) other coexisting malignant tumours; (2) severe respiratory, immune, haematologic, or cardiovascular system diseases; (3) incomplete data.

### Diagnostic criteria for PI

It must meet the following criteria (1) Newly developed cough, sputum production, or worsening symptoms of preexisting respiratory disease, with or without purulent sputum, chest pain, dyspnoea, haemoptysis, and fever (signs of pulmonary consolidation and/or wet rales heard on auscultation; peripheral blood white blood cell count > 10 × 10^9/L or < 4 × 10^9/L, with or without a left shift in the nucleus). (2) Chest imaging shows new patchy infiltrates, lobar or segmental consolidation, ground-glass opacities, or interstitial changes, with or without pleural effusion. By meeting any one of the above inclusion criteria and excluding tuberculosis, lung tumours, non-infectious interstitial lung disease, pulmonary oedema, atelectasis, pulmonary embolism, eosinophilic pneumonia, and vasculitis of the lungs, a clinical diagnosis can be established [10].

Given the overlapping clinical presentations, distinguishing true bacterial/fungal pneumonia from CIP is challenging. To address this, all cases of new or worsening pulmonary infiltrates were reviewed by a multidisciplinary adjudication committee comprising pulmonology, radiology, and oncology specialists. Patients were classified as having infection only if they met all of the following criteria: (i) Positive microbiological evidence from sputum, blood, or bronchoalveolar lavage fluid (BALF); or definitive clinical/radiographic resolution with antimicrobial therapy alone.(ii) Absence of classic CIP radiographic patterns (e.g., pure ground-glass opacities with subpleural sparing) without an alternative infectious etiology.(iii) No requirement for high-dose corticosteroid therapy (≥ 1 mg/kg prednisone equivalent) for respiratory decline [11–13].

Patients with a final MDT diagnosis of isolated CIP or concurrent CIP with superimposed infection were excluded from this analysis to preserve the homogeneity of the infectious cohort.

### Determination of positive pathogen test results

Positive detection of pathogens is based on the following criteria: (1) Bacteria (excluding mycobacteria): Positive results from blood and sputum cultures are considered positive. (2) *Mycobacterium tuberculosis*: Patients diagnosed as BALF positive by smear microscopy and acid-fast staining or an Xpert mycobacterium tuberculosis (MTB) test positive must meet the criteria of the official clinical practice guidelines of the American Thoracic Society/American College of Infectious Diseases/Centers for Disease Control and Prevention to be considered positive [14]. (3) Fungi: Patients diagnosed with fungal infection must meet at least one host factor, clinical feature, and fungal evidence, according to the diagnostic criteria of the European Organization for Research and Treatment of Cancer/European Society of Clinical Microbiology and Infectious Diseases [15]. (4) Viruses, mycoplasma, and chlamydia: Those who have a positive antigen test result by throat swab are considered positive.

Microorganisms detected only by mNGS at low read counts, or those representing common skin/oropharyngeal/nucleic acid extraction contaminants, were classified as contaminants/colonizers and excluded from the pathogen analysis, unless corroborated by clinical course and treatment response. All mNGS results were adjudicated by two independent infectious disease specialists and one clinical microbiologist, with discrepancies resolved by consensus. The final classification was made only after reviewing the complete clinical context.

### The definition of the positive mNGS test result of pathogen

The mNGS results were considered positive for a given pathogen when they met the following criteria: (1) Bacteria (excluding mycobacteria), viruses, Mycoplasma, and Chlamydia: The species-specific read count (sequence number) was at least tenfold higher than that of any other microorganism detected in the same sample, consistent with consensus recommendations [16]. This threshold was used to identify the dominant pathogenic signal among background and contaminating sequences. (2) Fungi: A sequence number ≥ 1 was required, and the diagnosis of fungal infection additionally required satisfaction of at least one host factor, one clinical feature, and one mycological evidence criterion, in accordance with the EORTC/MSGERC consensus definitions [15]. For Candida species specifically, stricter criteria were applied: Candida was considered a true pathogen only when detected at high read counts in BALF with supporting clinical, radiological, and microbiological evidence, and only after exclusion of colonization. Low-read-count detections in non-invasive specimens were classified as colonization/contaminants. (3) Mycobacteria: Due to the inherent difficulty of DNA extraction from mycobacterial cell walls, a sequence number ≥ 1 was considered positive for tuberculosis infection.

All mNGS results were adjudicated by two independent infectious disease specialists and one clinical microbiologist. The final classification of a microorganism as a true pathogen versus a contaminant/colonizer was determined only after comprehensive review of the complete clinical context, including host factors, clinical presentation, radiological findings, conventional microbiological results, and treatment response, as detailed in the “Diagnostic criteria for PI” section.

### Collection of patient’s data

Collect basic information of patients who meet the inclusion criteria. Including sex, age, smoking history, and underlying diseases. Clinical information included clinical symptoms, pathological type, tumour stage, number of chemotherapy sessions, and duration of ICI therapy combined with other lung diseases. And also collected the laboratory and imaging test results.

### Patient grouping

Grouping the patients into three groups. Chemotherapy with PI group: (1) Patients with newly diagnosed advanced NSCLC who developed PI during the course of chemotherapy alone were treated with the following chemotherapy regimens as pemetrexed combined with platinum- based (AP), and paclitaxel combined with platinum- based (TP). (2) Chemotherapy combined with ICIs with PI group: Patients with newly diagnosed advanced NSCLC who developed PI during the course of chemotherapy combined with ICIs were treated with the following chemotherapy regimens: AP or TP along with the ICIs as sintilimab, pembrolizumab and nivolumab. (3) Chemotherapy combined with ICIs without PI group: Patients with newly diagnosed advanced NSCLC who did not develop PI during the course of chemotherapy combined with ICIs were treated with the following chemotherapy regimens: AP or TP along with the ICIs as sintilimab, pembrolizumab and nivolumab.

### Intergroup comparisons

We analyzed the distribution characteristics of pathogens in the chemotherapy combined with ICIs with PI group and chemotherapy with PI group, and compared the bacterial communities of the infections between the two groups. Also, we analyzed the risk factors for PI between chemotherapy combined with ICIs with PI group and chemotherapy combined with ICIs without PI group.

### Statistical analysis

Applying SPSS 27.0 statistical software, the collected pathogenological and general patient data were entered into an excel database. Statistical analysis was conducted. Normally distributed quantitative data are represented as (x ± s). For nonnormally distributed data, the median (interquartile range) was used. Intergroup comparisons were made using the independent samples t test or Mann‒Whitney U test. For count data, rates (%) were used, and the χ^2^ test or Fisher’s exact probability method was applied. Multivariate analysis was conducted using logistic regression.To mitigate overfitting and confounding bias inherent in univariate P-value-driven selection, covariates for the multivariable logistic regression model were selected a priori based on clinical relevance and biological plausibility. Logistic regression analysis was used to screen for factors affecting the occurrence of secondary infections. Covariates were selected for multivariable analysis based on a combination of clinical relevance and univariate statistical association, while respecting the limitations of sample size to avoid model overfitting. *P* < 0.05 was considered statistically significant. In the multivariable logistic regression model, covariates were selected based on both statistical significance in univariate analysis (*P* < 0.05) and clinical relevance. The following variables were entered simultaneously into the model using the Enter method: disease stage (III vs. IV), number of ICIs treatment cycles (≥ 5 vs. <5), and lymphocyte count (as a continuous variable). Automated stepwise selection was not employed, in order to avoid known limitations including instability of variable selection and inflated type I error rates.

## Results

**Fig. 1:**
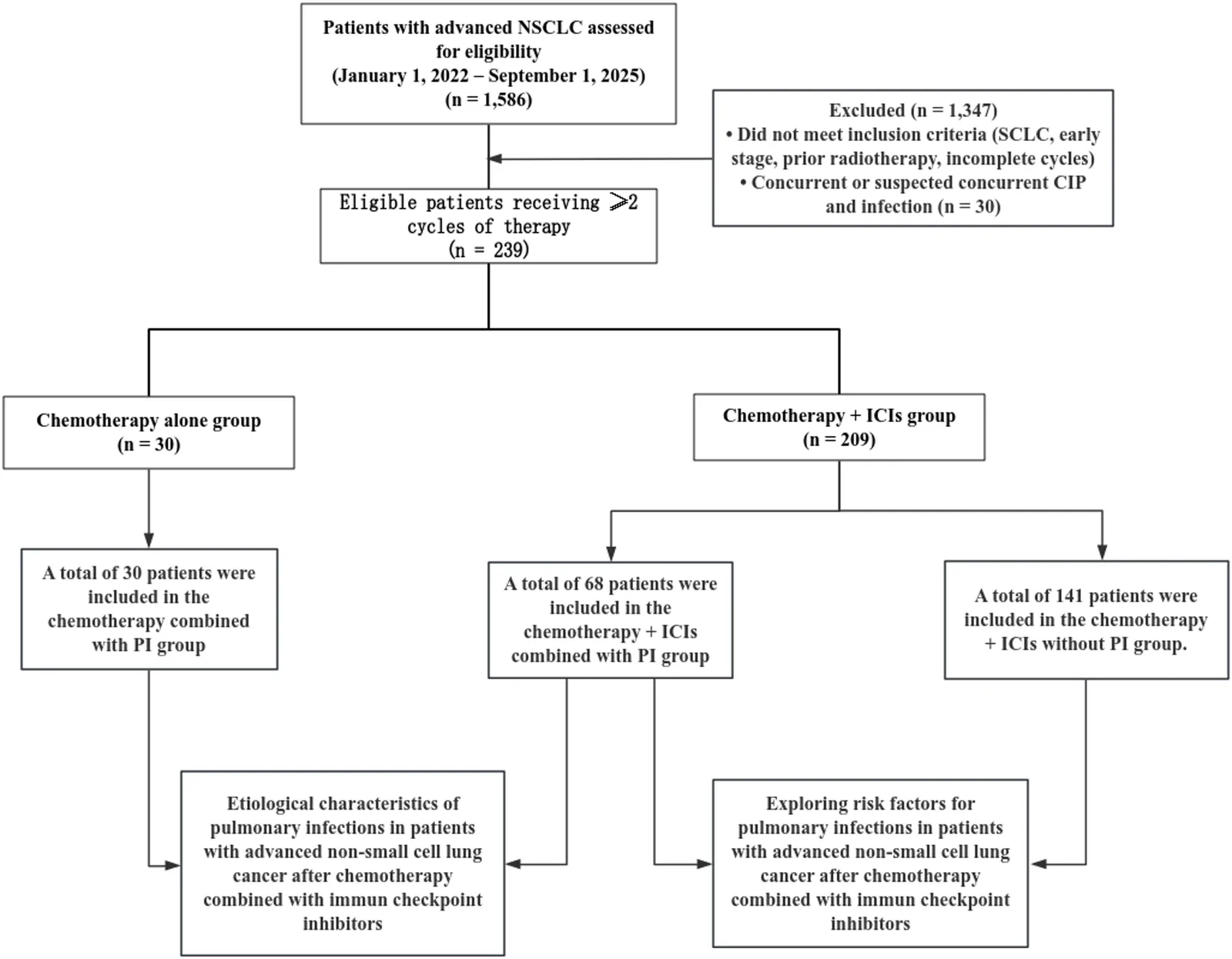
Research procedure diagram

In total, 1,586 cases of advanced NSCLC were included in this study. After screening, 239 patients met the inclusion criteria. In addition, chemotherapy combined with ICIs was used in 209 patients, including 68 (32.5%) patients assigned to the chemotherapy combined with ICIs with PI group and 141 (67.5%) patients assigned to the chemotherapy combined with ICIs without PI group. Moreover, 30 patients assigned to the chemotherapy with PI group. The three groups were included in this study, and the study steps are shown in Fig. 1.

### Clinical basic characteristics of patients in the chemotherapy combined with ICIs with PI group and chemotherapy with PI group

Statistical analysis was conducted on the baseline characteristics, All P values were greater than 0.05, indicating that there were no statistically significant differences in the characteristics between the two groups and that the baseline clinical characteristics of the patients in the two groups were consistent (See Table 1).

**Table 1:** Basic clinical characteristics of the chemotherapy combined with ICIs with PI group and chemotherapy combined with PI group

Characteristics	Chemotherapy combined with ICIs with PI group (*n* = 68)	Chemotherapy combined with PI group (*n* = 30)	χ^2^/Z Value	*P* Value
Age (year)	64.00 (59.25,67.75)	67.50 (61.00,72.25)	-1.783	0.075
Male (n(%))	62 (91.1)	27 (90.0)	0.000	1.000
Smoking history (n(%))	45 (66.1)	22 (73.4)	0.138	0.710
Hypertension (n(%))	22 (32.3)	9 (30.0)	0.053	0.817
COPD (n(%))	5 (7.4)	4 (13.3)	0.320	0.572
Symptom				
Diabetes mellitus (n(%))	10 (14.7)	3 (10.0)	0.096	0.757
Fever (n(%))	27 (39.7)	10 (33.3)	0.360	0.549
Shortness of breath, dyspnea (n(%))	28 (41.1)	13 (43.3)	0.040	0.842
Cough and sputum (n(%))	43 (63.2)	21 (70.0)	0.420	0.517
Imaging features				
Ground-glass opacities (n(%))	9 (13.2)	5 (16.6)	0.018	0.893
Patchy exudates (n(%))	58 (85.2)	23 (76.6)	1.081	0.299
Number of chemotherapy cycles applied ≥ 4 times (n(%))	52 (76.4)	15 (50.0)	0.754	0.385

Abbreviation: ICIs means immune checkpoint inhibitors; PI means pulmonary infection; COPD means chronic obstructive pulmonary disease. “n” represents the total number of patients in each group: the chemotherapy alone with PI group and the chemotherapy combined with ICIs with PI group

### Pathogen infection distribution

**Fig. 2:**
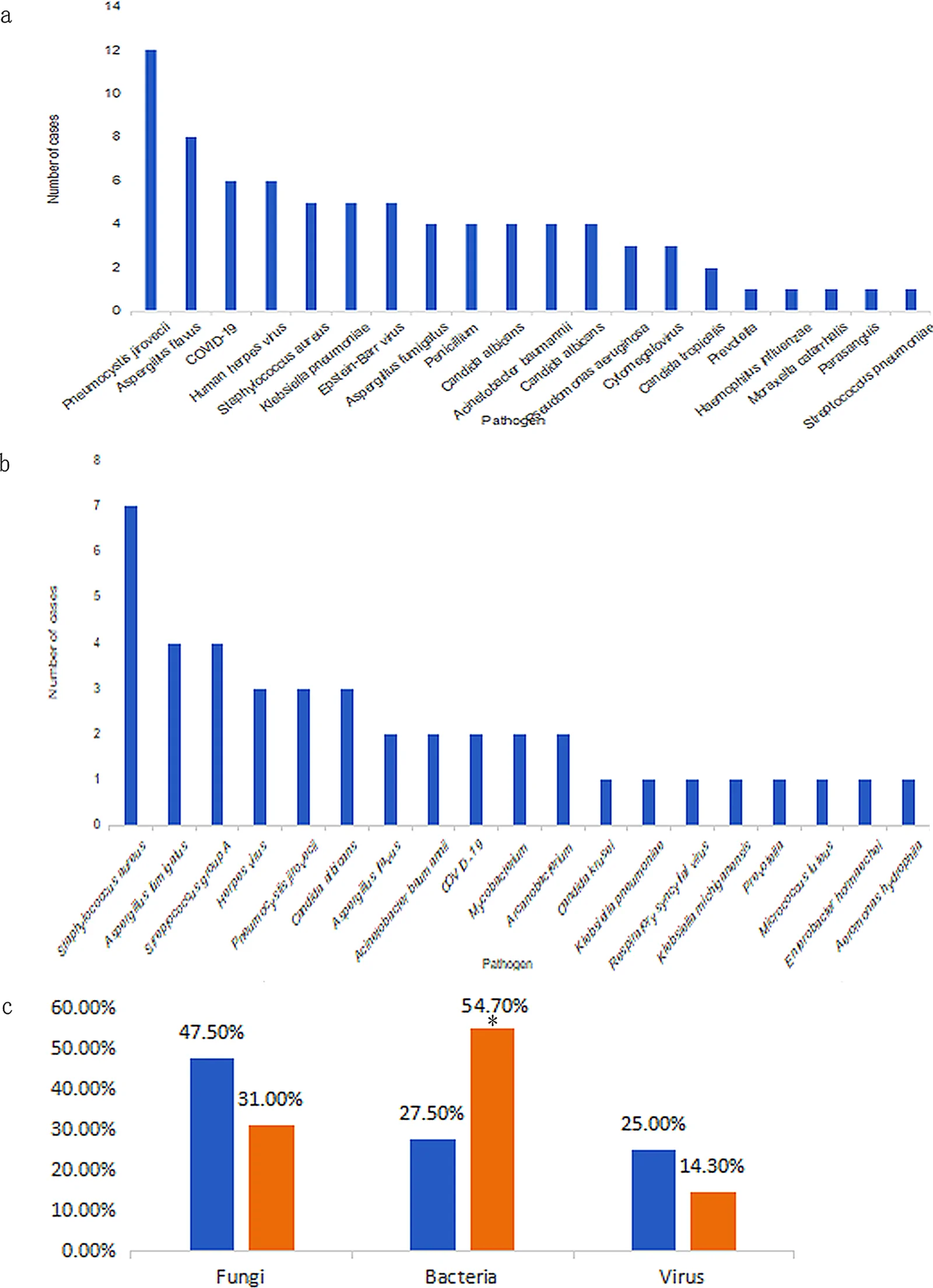
**a** Pathogen infection distribution in the chemotherapy combined with ICIs with PI group. The X-axis represents the names of pathogens in the chemotherapy combined with ICIs with PI group, and the Y-axis represents the number of cases of pathogen infections in the chemotherapy combined with ICIs with PI group. **b**. Pathogen distribution in chemotherapy combined with PI group. The X-axis represents the names of pathogens in the chemotherapy combined with PI group, and the Y-axis represents the number of cases of PI in the chemotherapy combined with PI group. **c**. Comparison of the pathogen distribution between the chemotherapy combined with ICIs with PI group and the chemotherapy combined with PI group. Blue represents the chemotherapy combined with ICIs with PI group, orange represents the chemotherapy combined with PI group; * indicates statistical significance; The X-axis represents three major pathogens: bacteria, fungi, and viruses, and the Y-axis represents the percentage of pathogens. (Abbreviation: ICIs means immune checkpoint inhibitors; PI means pulmonary infection)

In the chemotherapy combined with ICIs with PI group, fungi (47.50%) were the most common type of infection and mainly represented by *Pneumocystis jirovecii* (15.0%) and *Aspergillus flavus* (10.0%). In the chemotherapy combined with PI group, bacteria (54.70%) were present in the highest proportion, mainly *Staphylococcus aureus* (16.7%), *Streptococcus pneumoniae* (9.5%), and *Acinetobacter baumannii* (4.8%). The pathogen distribution characteristics of the two groups are shown in Table 2; Fig. 2.

**Table 2:** Pathogen distribution characteristics of the chemotherapy combined with ICIs with PI group and chemotherapy combined with PI group

Pathogen Types	Chemotherapy combined with ICIs with PI group(*n* = 80/68(%))	Chemotherapy combined with PI group(*n* = 42/30(%))
Fungi		
Pneumocystis jirovecii	12(15.0/22.1)	3(7.1/10.0)
Aspergillus flavus	8(10.0/11.8)	2(4.8/6.6)
Aspergillus fumigatus	4(5.0/5.9)	4(9.5/13.3)
Penicillium	4(5.0/5.9)	0(0.0/0.0)
Candida albicans	4(5.0/5.9)	3(7.1/10.0)
Candida glabrata	4(5.0/5.9)	0(0.0/0.0)
Candida tropicalis	2(2.5/2.9)	0(0.0/0.0)
Candida krusei	0(0.0/0.0)	1(2.4/3.3)
Bacteria		
Staphylococcus aureus	5(6.3/7.4)	7(16.7/23.3)
Streptococcus pneumoniae	1(1.3/1.5)	4(9.5/13.3)
Streptococcus pyogenes	1(1.3/1.5)	0(0.0/0.0)
Klebsiella pneumoniae	5(6.3/7.4)	2(4.8/6.6)
Mycobacterium tuberculosis	0(0.0/0.0)	2(4.8/6.6)
Acinetobacter baumannii	4(5.0/5.9)	2(4.8/6.6)
Haemophilus influenzae	1(1.3/1.5)	0(0.0/0.0)
Pseudomonas aeruginosa	3(3.8/4.4)	0(0.0/0.0)
Escherichia coli	0(0.0/0.0)	0(0.0/0.0)
Enterobacter cloacae	0(0.0/0.0)	1(2.4/3.3)
Staphylococcus warneri	0(0.0/0.0)	2(4.8/6.6)
Micrococcus luteus	0(0.0/0.0)	1(2.4/3.3)
Aeromonas hydrophila	0(0.0/0.0)	1(2.4/3.3)
Prevotella	1(1.3/1.5)	1(2.4/3.3)
Moraxella catarrhalis	1(1.3/1.5)	0(0.0/0.0)
Virus		
Pneumocystis johnensii	6(7.5/8.9)	3(7.1/10.0)
COVID-19	6(7.5/8.9)	2(4.8/6.6)
Cytomegalovirus	3(3.8/4.4)	0(0.0/0.0)
Syncytium virus	0(0.0/0.0)	1(2.4/3.3)
EB virus	5(6.3/7.4)	0(0.0/0.0)

Percentages in Tables are calculated based on the total number of pathogen isolates rather than the number of patients. (“n” represents the total number of infectious pathogens / total patients in each group: the chemotherapy alone with PI group and the chemotherapy combined with ICIs with PI group)

### Comparison of the distribution of pathogens

Between the chemotherapy combined with ICIs with PI group and the chemotherapy with PI group revealed that the prevalence of bacterial infection (χ^2^ = 8.792; *P* = 0.003), had significantly differ (See Table 3; Fig. 2). This study further statistically compared the fungal pathogens between the chemotherapy combined with ICIs with PI group and the chemotherapy with PI group. The results revealed that the proportions of *Pneumocystis jirovecii* (χ^2^ = 0.442, *P* = 0.506) and *Aspergillus* (χ^2^ = 0.077, *p* = 0.782), and *Candida* (χ^2^ = 0.032, *P* = 0.858) did not significantly differ between the two groups of PI (See Table 4).

**Table 3:** Distribution of pathogen characteristics in the chemotherapy combined with ICIs with PI and chemotherapy combined with PI group

Pathogen Types	Chemotherapy combined with ICIs with PI group (*n* = 80/68(%))	Chemotherapy combined with PI group (*n* = 42/30(%))	χ^2^ Value	*P* Value
Fungi	38 (47.5/55.9)	13 (31.0/43.3)	3.100	0.078
Bacteria	22 (27.5/32.3)	23 (54.7/76.7)	8.792	0.003
Virus	20 (25.0/29.4)	6 (14.3/20.0)	1.885	0.170

Data are presented as: number of pathogen isolates/total isolates (isolate-based %) / number of total patients (patient-based %). Percentages calculated based on pathogen isolates reflect the full pathogen spectrum including polymicrobial infections, while percentages based on patients reflect the proportion of patients harboring each pathogen type. (“n” represents the total number of infectious pathogens / total patients in each group: the chemotherapy alone with PI group and the chemotherapy combined with ICIs with PI group)

**Table 4:** Analysis of the proportion of fungal pathogens in the chemotherapy combined with ICIs with PI group and chemotherapy combined with PI group

Fungi pathogen types	Chemotherapy combined with ICIs with PI group(*n* = 68)	Chemotherapy combined with PI group(*n* = 30)	Χ^2^/Z Value	*P* Value
*Pneumocystis jirovecii* (n (%))	12 (17.6)	3 (10.0)	0.442	0.506
*Aspergillus* (n (%))	12 (17.6)	6 (20.0)	0.077	0.782
*Penicillium* (n (%))	4 (5.9)	0 (0.0)	-	-
*Candida* (n (%))	10 (14.7)	4 (13.3)	0.032	0.858

Abbreviation: ICIs means immune checkpoint inhibitors; PI means pulmonary infection. “n” represents the total number of patients in each group: the chemotherapy alone with PI group and the chemotherapy combined with ICIs with PI group

### Statistical analysis of patients with mixed infections

There were 29 cases of mixed infections in the chemotherapy combined with ICIs with PI group. This included 4 cases (13.8%) of mixed bacterial, fungal, and viral infections; 12 cases (41.4%) of bacterial infections combined with fungal infections;3 cases (10.3%) of bacterial infections combined with viral infections; and 8 cases (27.5%) of fungal infections combined with viral infections.

**Fig. 3:**
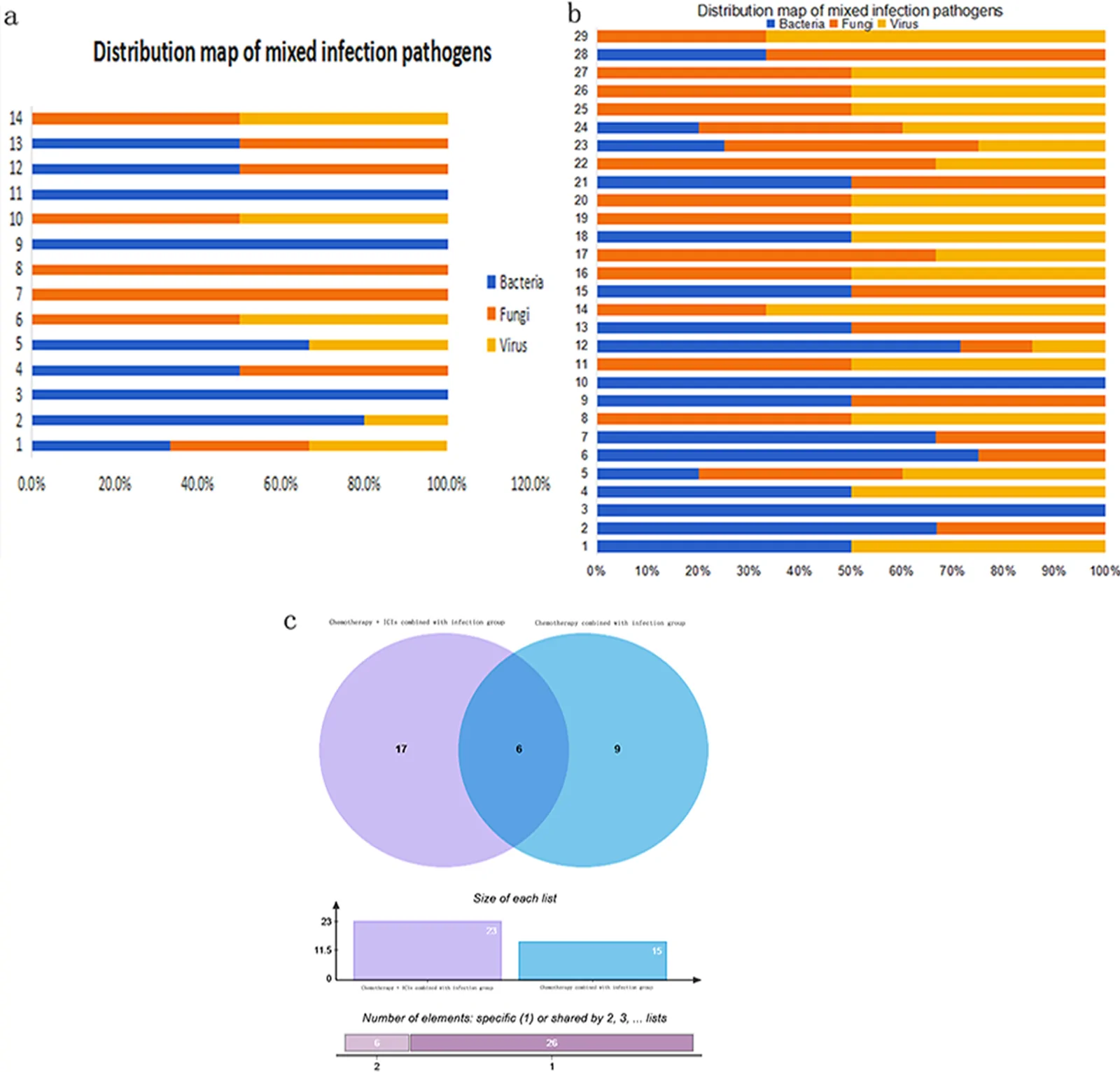
(**a**) Proportion of mixed infections in the chemotherapy combined with ICIs with PI group, Blue represents bacteria, orange represents fungi, yellow represents viruses. The X-axis indicates the percentage of pathogens, and the Y-axis represents the encoding of mixed infections in each case. (**b**) Proportion of mixed infections in the chemotherapy combined with PI group. Blue represents bacteria, orange represents fungi and yellow represents viruses. The X-axis indicates the percentage of pathogens, and the Y-axis represents the encoding of mixed infections in each case. (**c**) Venn diagram of mixed infections, purple represents the number of pathogens in all mixed infection cases for the chemotherapy combined with ICIs with PI group, blue represents the number of pathogens in all mixed infection cases for the chemotherapy combined with PI group, and the intersection part as well as the number of pathogens that are common infections between the two. Size of each list: The X-axis represents the name of each group, and the Y-axis represents the number of pathogen types. (Abbreviation: ICIs means immune checkpoint inhibitors; PI means pulmonary infection)

In the chemotherapy with PI group, there were 14 cases of mixed infections. This included 1 case (7.1%) of mixed bacterial, fungal, and viral infections; 3 cases (21.5%) of bacterial infections combined with fungal infections; 2 cases (14.3%) of bacterial infections combined with viral infections; and 3 cases (21.5%) of fungal infections combined with viral infections. This finding indicates to some extent that fungal infections combined with viral infections are more common among mixed infections (Table 5). For a more intuitive presentation, a statistical chart is shown in Fig. 3.

Comparing the proportions of mixed infections involving both combinations of chemotherapy and PI, the results showed that P values were greater than 0.05 for both cases, indicating no statistical difference between the two groups. Further analysis of the data on mixed infections involving fungi revealed that the proportion of such infections in patients treated with chemotherapy plus ICIs and PI was 82.7%, while it was 50.0% in those treated with chemotherapy and PI alone. Chi-square analysis of the four-cell table showed a statistically significant difference in fungal infection rates between the two groups (Fisher’s exact test, *p* = 0.035). This suggests that chemotherapy combined with ICIs significantly increases the risk of fungal infections in patients with advanced NSCLC.(Table 5).

**Table 5:** Mixed infection analysis table

	Chemotherapy combined with ICIs with PI group(*n* = 29)	Chemotherapy combined with PI group(*n* = 14)	Fisher’s exact probability method (*p*-value, two-sided)
Combined fungal mixed infection(n(%))	24(82.7)	7(50.0)	0.035
Non-fungal mixed infection(n(%))	5(17.3)	7(50.0)	
Bacteria+Fungi+Virusn(n (%))	4(13.8)	1(7.1)	1.000
Bacteria+Fungin(n (%))	12(41.4)	3(21.5)	0.308
Bacteria+Virusn(n (%))	3(10.3)	2(14.3)	1.000
Fungi+Virusn(n (%))	8(27.5)	3(21.5)	1.000

“n” = total number of patients with mixed infections in the respective group

### Timing of PI occurrence in the chemotherapy combined with ICIs with PI group and the chemotherapy with PI group

**Fig. 4:**
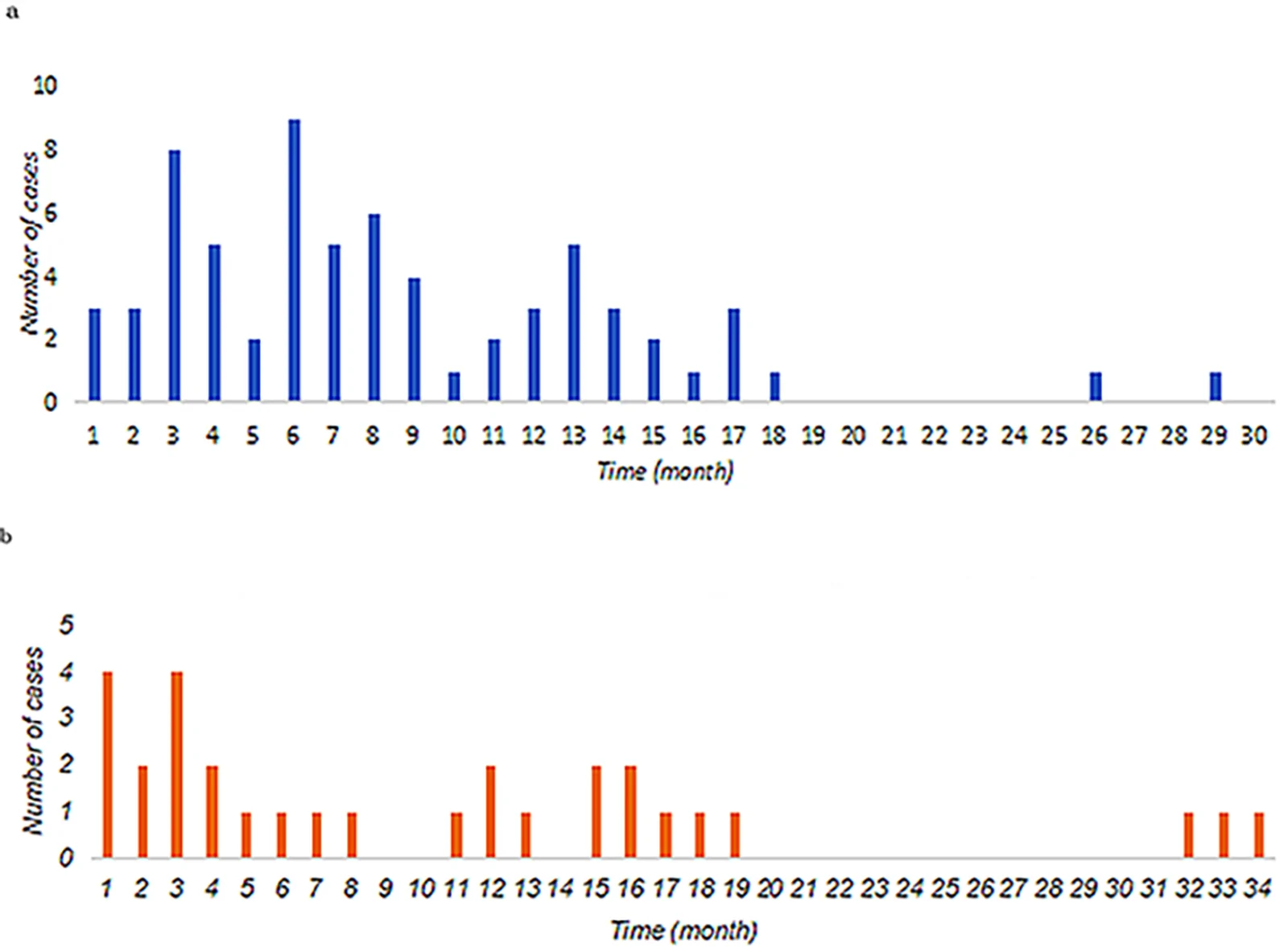
(**a**) Timing of infection in the chemotherapy combined with ICIs with PI group. The X-axis represents the time of infection occurrence in the chemotherapy combined with ICIs with PI group, and the Y-axis represents the number of infections occurring at corresponding times in the chemotherapy combined with ICIs with PI group. (**b**) Infection duration in the chemotherapy combined with PI group. The X-axis represents the time of infection occurrence in the chemotherapy combined with PI group, and the Y-axis represents the number of infections occurring at corresponding times in the chemotherapy combined with PI group. (Abbreviation: ICIs means immune checkpoint inhibitors; PI means pulmonary infection)

Among the patients in the chemotherapy combined with ICIs with PI group, 30 developed PI within 6 months of diagnosis, 51 developed PI within 12 months, and 66 developed pulmonary infections within 18 months. The greatest number of PI occurred in the sixth month after diagnosis (9 cases), followed by the third month (8 cases). Overall, the number of PI fluctuated across months, but most pulmonary infections occurred within 18 months (97.1%). The distribution of the frequency of PI at different times is shown in Table 6; Fig. 4.

In patients with the chemotherapy with PI group, 14 developed PI within 6 months after diagnosis, 19 developed PI within 12 months, and 27 developed PI within 18 months. The highest number of PI cases occurred in the first and third months after diagnosis, reaching a total of 4 cases. Overall, the number of PI cases fluctuated across different months, but most PI occurred within 18 months (90.0%). The frequency distribution of PI at different times is shown in Table 6; Fig. 4.

**Table 6:** Statistical analysis of the duration of pulmonary infection in chemotherapy combined with ICIs with PI group and chemotherapy combined with PI group

Time of infection (month)	Chemotherapy combined with ICIs with PI group (*n* = 68(%))	Chemotherapy combined with PI group (*n* = 30(%))
1	3 (4.4)	4 (13.3)
2	3 (4.4)	2 (6.7)
3	8 (11.8)	4 (13.3)
4	5 (7.4)	2 (6.7)
5	2 (2.9)	1 (3.3)
6	9 (13.2)	1 (3.3)
7	5 (7.4)	1 (3.3)
8	6 (8.8)	1 (3.3)
9	4 (5.9)	0 (0.0)
10	1 (1.5)	0 (0.0)
11	2 (2.9)	1 (3.3)
12	3 (4.4)	2 (6.7)
13	5 (7.4)	1 (3.3)
14	3 (4.4)	0 (0.0)
15	2 (2.9)	2 (6.7)
16	1 (1.5)	2 (6.7)
17	3 (4.4)	1 (3.3)
18	1 (1.5)	1 (3.3)
19	0 (0.0)	1 (3.3)
20	0 (0.0)	0 (0.0)
21	0 (0.0)	0 (0.0)
22	0 (0.0)	0 (0.0)
23	0 (0.0)	0 (0.0)
24	0 (0.0)	0 (0.0)
25	0 (0.0)	0 (0.0)
26	1 (1.5)	0 (0.0)
27	0 (0.0)	0 (0.0)
28	0 (0.0)	0 (0.0)
29	1 (1.5)	0 (0.0)
≥ 30	0(0.0)	3 (10.0)

“n” represents the total number of patients in each group: the chemotherapy alone with PI group and the chemotherapy combined with ICIs with PI group

### Risk factors

**Fig. 5:**
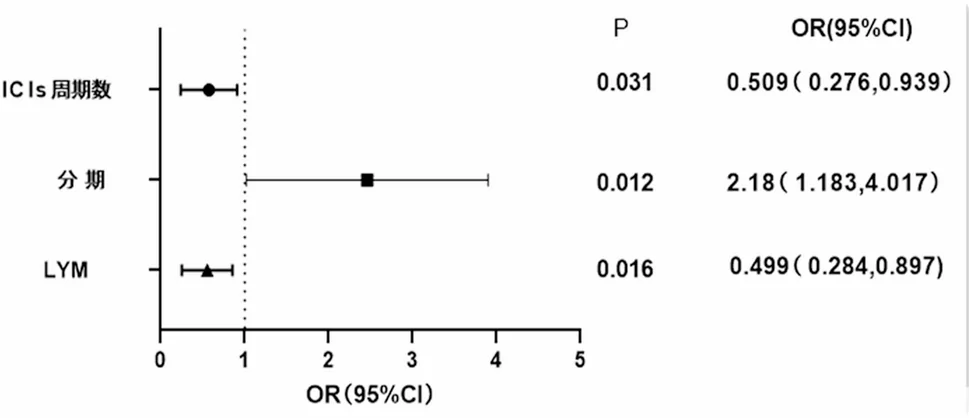
Multivariate analysis of risk factors for PI in patients with advanced NSCLC after treatment with chemotherapy combined with ICIs, where the X-axis represents odds ratio (OR) and the Y-axis represents risk factors

To compare the clinical characteristics and data between the chemotherapy combined with ICIs with PI group and the chemotherapy combined with ICIs without PI group, Univariate analysis showed that statistically significant differences between the two groups in terms of stage IV disease (χ^2^ = 7.943, *P* = 0.005), the number of patients with ≥ 5 cycles of immunotherapy (χ^2^ = 5.820, *P* = 0.016), low lymphocyte (LYM) counts (Z=-2.69; *P* = 0.007). As shown in Table 7. Three variables—disease stage, ICIs treatment cycle number, and lymphocyte count—were entered simultaneously into the multivariable logistic regression model using the Enter method. The results revealed that LYM (OR 0.499, *P* = 0.016), number of cycles of immunotherapy ≥ 5 (OR 0.509, *P* = 0.031), and stage IV disease (OR 2.18, *P* = 0.012) were significantly associated with PI (see Table 8; Fig. 5).

**Table 7:** Clinical characteristics and univariate analysis of the chemotherapy combined with ICIs with PI group and chemotherapy combined with ICIs without PI group

Outcome measures	Chemotherapy combined with ICIs with PI group(*n* = 68)	Chemotherapy combined with ICIs without PI group (*n* = 141)	χ^2^ /Z Value	*p* Value
**Sex (n(%))**			0.701	0.402
Male	62(91.1)	123(87.3)		
Female	6(8.8)	18(12.7)		
**Age (n(%))**	64.0(59.2,69.0)	65.0(60.0,69.0)	-1.387	0.165
Smoking history (n(%))			0.349	0.555
Yes	46(67.6)	101(71.6)		
No	22(32.3)	40(28.3)		
**Histology (n(%))**			0.252	0.922
Adenocarcinoma	37(54.4)	73(51.7)		
Squamous carcinoma	29(42.6)	64(45.3)		
Other types	2(2.9)	4(2.8)		
**Stage (n(%))**			7.943	0.005
III(b ~ c)	36(52.9)	46(32.6)		
IV(a ~ c)	32(47.0)	95(67.3)		
**Hypertension history (n(%))**			2.281	0.131
Yes	22(32.3)	61(43.2)		
NO	46(67.6)	80(56.7)		
**COPD history (n(%))**			0.005	0.945
Yes	5(7.3)	10(7.1)		
NO	63(92.6)	131(92.9)		
**Diabetes history (n(%))**			0.181	0.671
Yes	10(14.7)	24(17.0)		
NO	58(85.3)	117(82.9)		
**Chemotherapy cycle number (n(%))**			0.098	0.754
≥ 4	52(76.4)	105(74.5)		
<4	16(23.6)	36(25.5)		
**ICIs treatment cycle number (n(%))**			5.820	0.016
≥ 5	35(51.5)	48(34.1)		
<5	33(48.5)	93(65.9)		
**Chemotherapy regimen (n(%))**			5.295	0.259
Paclitaxel combined with platinum-based	32(47.0)	69(48.9)		
Pemetrexed combined with platinum-based	22(32.3)	58(41.1)		
**ICIs (n(%))**			2.746	0.434
Sintilimab	28(41.2)	73(51.7)		
Tislelizumab	19(27.9)	37(26.2)		
Pembrolizumab	17(25.0)	25(17.7)		
Other types ICIs	4(5.8)	6(4.2)		
**Clinical diagnosis (n(%))**			-	-
Shortness	28(41.2)	0	-	-
Dyspnea	43(63.2)	0	-	-
Cough	27(39.7)	0	-	-
**laboratory results (n(%))**				
White blood cell count	7.18(5.87, 9.24)	8.08(6.43, 9.42)	-1.636	0.102
Neutrophil count	5.22(3.93, 6.72)	5.73(4.39, 7.10)	-1.529	0.126
Lymphocyte count	1.33±0.53	1.52(1.26, 1.82)	-2.69	0.007
Eosinophil count	0.08(0.04, 0.21)	0.12(0.06, 0.21)	-1.399	0.162
Lactate dehydrogenase	216.0(180.2, 253.0)	205.0(173.0, 243.0)	-1.232	0.218
Albumin	38.73±4.73	39.4(36.35, 41.9)	-0.393	0.694
Albumin globulin Ratio	1.37±0.26	1.36(1.18, 1.58)	-0.095	0.924
Neutrophil-to-Lymphocyte ratio	3.98(2.84, 5.82)	3.92(2.85, 5.05)	-0.781	0.435
Carcinoembryonic antigen	5.27(3.11, 10.86)	6.14(3.30, 21.98)	-1.348	0.178
Neutrophil percentage	71.58±9.3614	71.87(66.14,76.36)	-0.181	0.857
Lymphocyte percentage	18.97±8.37	19.05(15.85,23.71)	-1.069	0.285
Eosinophil percentage	1.40(0.66,2.21)	1.63(0.73,3.03)	-0.948	0.343

Abbreviation: ICIs means immune checkpoint inhibitors; PI means pulmonary infection; COPD means chronic obstructive pulmonary disease. “n” represents the total number of patients in each group: the chemotherapy combined with ICIs with PI group and Chemotherapy combined with ICIs without PI group

**Table 8:** Multivariate analysis of the chemotherapy combined with ICIs with PI group and chemotherapy combined with ICIs without PI group

Risk factors	B	SE	Wald	*P*	OR	95%CI
LYM	-0.695	0.288	5.801	0.016	0.499	(0.284, 0.897)
Stage = IV	0.779	0.312	6.241	0.012	2.18	(1.183, 4.017)
ICIs treatment cycle number ≥ 5	-0.674	0.312	4.68	0.031	0.509	(0.276, 0.939)

## Discussion

Chemotherapy is the main treatment for patients with advanced lung cancer. With the continuous development of antitumour drugs, ICIs have been widely used to treat advanced lung cancer [2]. In the treatment of lung cancer, the incidence of pulmonary infections is relatively high, especially among elderly patients with advanced lung cancer, owing to a decline in immune function [17]. This study aimed to explore the pathogen distribution characteristics and risk factors for pulmonary infections in patients with advanced NSCLC after receiving chemotherapy combined with ICIs.

This study compared and analysed the pathogenic characteristics of PI in patients with advanced NSCLC after receiving chemotherapy combined with ICIs and revealed that PI was more common in the group with concurrent infections, mainly *Pneumocystis jirovecii* and *Aspergillus* species. Therefore, during the clinical diagnosis and treatment of patients with advanced lung cancer, trimethoprim-sulfamethoxazole (TMP-SMX) can be appropriately used to prevent *Pneumocystis jirovecii* pneumonia (PJP), and monitoring should be strengthened with timely preventive medication. *Pneumocystis jirovecii* is an atypical opportunistic fungal pathogen that exhibits genetic diversity; it is more susceptible to infection in immunocompromised or immunodeficient patients, especially HIV-infected individuals [18]. In recent years, studies have reported that the prevalence, incidence, and mortality rates of *Pneumocystis jirovecii* colonization and PJP among non- human immunodeficiency virus (HIV) - infected individuals have been increasing annually [18]. *Aspergillus* is an opportunistic pathogen that widely exists in nature and can parasitize and colonize the respiratory tract. Neutrophils and macrophages can phagocytose fungal spores and hyphae when immune function is normal; however, when immunity is reduced, pulmonary aspergillosis is prone to occur [19]. The incidence rate continues to increase with the use of immunosuppressants and broad-spectrum antibiotics, characterized by recurrent disease, variable symptoms, and prolonged nonhealing, making it an important factor in the death of immunosuppressed patients [20]. Patients with advanced lung cancer who are receiving ICIs face a higher risk of pulmonary fungal infections, and sufficient attention should be given to clinical treatment to take corresponding preventive and therapeutic measures.

In recent years, with the advancement of detection technology, several studies have shown that bacteria, fungi, viruses, and *Mycobacterium tuberculosis* are common pathogens in lung cancer patients with infection [21–24]. This study assessed the distribution of common pathogens in patients with advanced NSCLC who developed PI after receiving chemotherapy combined with ICIs, providing diagnostic and treatment evidence for patients receiving comprehensive therapy.

This study revealed that low lymphocyte count and stage IV disease were associated with an increased likelihood of PI, and ≥ 5 cycles of immunotherapy was associated with a reduced likelihood of PI in patients with advanced NSCLC receiving chemotherapy combined with ICIs. However, it must be emphasized that these associations were identified in a multivariable model that did not adjust for several critical confounders, most notably baseline corticosteroid use—the single most important risk factor for Pneumocystis jirovecii and other opportunistic fungal infections. The unmeasured influence of corticosteroid exposure may substantially confound the observed associations. Therefore, these findings should be interpreted with considerable caution and regarded as hypothesis-generating rather than definitive. They require validation in prospective studies with rigorous collection and adjustment for corticosteroid use and other relevant confounders. The lymphocyte count is an indicator that reflects the body’s immune function and indicates the body’s ability to defend against pathogens, especially as a core quantitative indicator for fighting fungi, viruses, and bacteria [25]. A decrease in lymphocyte count predicts an increased risk of infection, especially with opportunistic pathogens such as herpes viruses, *Pneumocystis jirovecii*, *Candida*, and *Mycobacterium tuberculosis* [26–28]. When ICIs are used in patients with advanced lung cancer, the patient’s immune system is further weakened, reducing its defence against pathogens. Therefore, monitoring the lymphocyte count is particularly important when ICIs are used to treat patients with advanced NSCLC.

In this study, stage IV disease was found to be an factors independently associated with an increased likelihood of PI in patients with advanced NSCLC, which is consistent with the findings of multiple studies [29]. The reason is that the tumour stage of patients with advanced NSCLC is closely related to the occurrence of pulmonary infections. The later the tumour stage is, the higher the likelihood of PI. Patients in late stages have a large tumour burden, which can directly invade the airways and lung tissue, leading to airway obstruction, atelectasis, loss of local defence function, and easier colonization and infection by pathogens. Patients with advanced tumours often have poor overall nutritional status and cachexia, which further weakens the body’s immune function, increasing patient susceptibility to infection [30].

While these findings are not entirely novel, the incremental value of our research lies in three key aspects: First, we provide updated evidence specific to the Chinese population, addressing the gap in region-specific data amid known ethnic disparities in lung cancer epidemiology. Second, our study focuses on the contemporary first-line standard of care (ICIs combined with chemotherapy), while prior evidence primarily derives from chemoradiotherapy cohorts [31], our data extend these observations to the chemotherapy-immunotherapy combination setting. Third, our variable selection strategy, which balanced statistical significance with clinical relevance, ensures the robustness and clinical interpretability of our risk model.

Patients with advanced lung cancer often need to use ICIs for a long time during the treatment of malignant tumours. ICIs therapy activates the body’s own immune system to kill tumour cells, but the therapy may also cause immune-related adverse reactions during treatment, among which PI is relatively serious. There is no clear explanation for the relationship between the number of immunotherapy cycles and the occurrence of PI. One study revealed that patients who received five or more cycles had greater intraoperative blood loss [32]. thus, this study, 5 cycles of ICIs were selected for analysis.

This study demonstrates a significant association between the number of ICIs cycles and the risk of pulmonary infections in patients with advanced NSCLC receiving chemoimmunotherapy. Specifically, completion of ≥ 5 ICIs cycles was associated with a reduced likelihood of pulmonary infections, a finding consistent with prior research [33]. This finding is susceptible to immortal time bias, as completing ≥ 5 cycles inherently selects for patients with better prognosis and longer survival. Therefore, this association should not be interpreted as a causal protective effect. Notably, in our cohort, no patient discontinued ICIs treatment due to pulmonary infection; however, immortal time bias through other pathways (early progression, death) remains a concern. These observations may be attributed to several underlying mechanisms. First, patients who complete ≥ 5 cycles of ICIs therapy typically exhibit sustained therapeutic responses and have not discontinued treatment due to severe immune-related adverse events. Consequently, their tumor burden remains better controlled, thereby mitigating the risk of obstructive pneumonia and secondary infections resulting from central airway obstruction by tumors [33]. Second, effective long-term ICIs treatment may ameliorate systemic chronic inflammation and attenuate the deleterious effects of the tumor microenvironment on the airway barrier [34]. Therefore, within the context of combined ICIs-chemotherapy regimens, undergoing ≥ 5 cycles of ICIs treatment serves as a surrogate marker for clinical benefit, indirectly reflecting a reduced susceptibility to pulmonary infections. These findings suggest that for patients who tolerate the regimen and maintain a durable response, completing the full course of ICIs treatment is advantageous for infection prophylaxis.

In recent years, ICIs therapy has become the main treatment for patients with advanced NSCLC. It activates the body’s own immune system to kill tumour cells, but it may also cause adverse immune-related reactions during treatment, among which PI is relatively serious. Understanding the risk factors, using accurate diagnostic methods, and formulating effective treatment strategies are crucial for improving the quality of life and prognosis of patients with advanced NSCLC. During clinical management, it is important to dynamically monitor patient lymphocyte levels to increase immune defence; timely use of TMP-SMX can prevent PJP, strengthen monitoring, and provide preventive medication when appropriate. This will improve the prognosis and treatment of patients with advanced NSCLC and prolong their survival.

Several important limitations of this study warrant careful consideration when interpreting the results. First, this is a single-centre retrospective study, and the data are only from a specific population (patients of our hospital); thus, the generalizability of its conclusions needs to be verified by more external data. Second, not all evidence of pathogens causing PI was obtained from high-throughput gene sequencing results, and some cases were based on sputum culture results, which may lead to certain heterogeneity. Third, it is important to acknowledge that classifying patients into either a chemotherapy-only group or a chemotherapy plus immunotherapy group based solely on their medication use has certain limitations. This factor may affect our interpretation of the study results, especially when comparing the distribution of pathogens between the two groups. Although we compared the baseline data, no statistically significant differences were found (all P-values were greater than 0.05). However, the lack of statistical significance does not prove the absence of clinical imbalances, particularly given the relatively small sample size. The hypothesis tests used to detect differences in baseline characteristics were designed to identify disparities rather than prove equivalence between the two treatment approaches. These test results are largely influenced by statistical power. Therefore, it is impossible to rule out confounding factors related to the different treatment methods.

Fourth, our analysis was unable to incorporate several key clinical variables that are known to strongly influence infection risk, including ECOG performance status, corticosteroid use, and prior antibiotic exposure. The absence of these variables represents a significant source of potential residual confounding. ECOG performance status is a critical determinant of both treatment eligibility and overall vulnerability to infectious complications. Patients with poorer performance status may be preferentially selected for less intensive regimens, and simultaneously harbor a higher intrinsic risk of infection independent of treatment type. Consequently, any observed difference in infection rates between groups could be partially or wholly attributable to this baseline imbalance rather than to the addition of immunotherapy. Similarly, corticosteroid use—frequently employed for symptom management or immune-related adverse event prophylaxis—has profound immunomodulatory effects that can increase susceptibility to a broad range of pathogens, including opportunistic infections. Without adjusting for corticosteroid exposure, we cannot exclude the possibility that differential use between groups confounds our infection-related endpoints. Notably, corticosteroid use represents the single most important confounder for fungal infections in this context, particularly Pneumocystis jirovecii pneumonia. Unfortunately, detailed information regarding the duration and dosage of glucocorticoid use was not systematically recorded in the medical records of a substantial proportion of our retrospective cohort. While some records contained fragmentary mentions of corticosteroid administration, the documentation was too incomplete and inconsistent to permit reliable extraction of even a binary variable (yes/no) for corticosteroid use in the preceding 3 months. We fully acknowledge that this represents a critical limitation, and the absence of this variable may significantly confound the associations identified in our risk factor model. This further underscores the importance of interpreting our multivariate findings with substantial caution.

Furthermore, prior antibiotic exposure is a well-established risk factor for subsequent colonization and infection with multidrug-resistant organisms, and it substantially alters the baseline microbiota and pathogen distribution landscape. The lack of data on antecedent antibiotic use limits our ability to determine whether the observed differences in pathogen spectrum—such as a potentially higher proportion of resistant Gram-negative bacteria in one group—reflect a true biological effect of chemo-immunotherapy or merely differences in antibiotic pressure between the groups. This is particularly pertinent to the interpretation of pathogen distribution comparisons, which are more sensitive to such ecological confounders than crude infection rate analyses.

Collectively, these unmeasured confounders may bias the results in unpredictable directions. For instance, if patients receiving chemo-immunotherapy had systematically better performance status, lower corticosteroid exposure, and less prior antibiotic use, the apparent protective or neutral association of immunotherapy on infection risk could be overestimated. Conversely, if the immunotherapy group had a higher burden of these risk factors, any true protective association might be attenuated or masked. Given this uncertainty, the findings of this study—especially those pertaining to infection incidence and pathogen distribution—should be interpreted as hypothesis-generating and warrant validation in prospective cohorts with rigorous collection and adjustment for these critical variables. The risk factor analysis for pulmonary infection was limited to the chemo-immunotherapy cohort, and the chemotherapy-only group was not included. This design precludes a formal assessment of whether the addition of ICIs itself is an independent risk factor for infection. This approach was adopted because the primary objective was to identify predictors specific to the chemo-immunotherapy population, and because the decision to administer ICIs is heavily confounded by indication, which cannot be fully adjusted for in a retrospective analysis. We acknowledge this as a limitation, and future prospective studies are needed to address this question directly.

Additionally, owing to individual subjective differences in patient selection, the cases included in this study did not undergo measurements of peripheral blood lymphocytes and other immune function indicators, resulting in insufficient statistical power when their relationship with the development of pulmonary infections was analysed. In some samples, the records of ECOG performance status scores were incomplete; there was also a lack of detailed information regarding the duration and dosage of glucocorticoid use. Additionally, records of antibiotic usage were insufficient. Therefore, these samples were not included in the analysis. Future studies should adopt a prospective, multicentre design, include more comprehensive variables (such as immune function indicators), and use a larger sample size to verify and extend the findings of this study.

## Conclusions

In this observational study, bacterial infections were observed more frequently in patients receiving chemotherapy alone, while fungal infections constituted the predominant pathogens among those treated with chemotherapy combined with ICIs. Low lymphocyte count and stage IV disease were associated with an increased risk of PI in patients receiving chemotherapy combined with ICIs, whereas receiving ≥ 5 cycles of ICIs was associated with a reduced risk. However, this association is susceptible to immortal time bias, as completing ≥ 5 cycles inherently selects for patients with better prognosis and longer survival. Given the non-randomized design and the potential for residual confounding, these associations should be interpreted as hypothesis-generating. The designation of ‘independent risk/protective factors’ should be viewed with caution pending validation in prospective studies.

## Data Availability

Data is provided within the manuscript. The data that support the findings of this study are available from the corresponding author upon reasonable request.

## Abbreviations

NSCLC: Non-small cell lung cancer
ICIs: Immune checkpoint inhibitors
PI: Pulmonary infection
CIP: Checkpoint inhibitor- related pneumonia
BALF: Bronchoalveolar lavage fluid
MTB: Mycobacterium tuberculosis
mNGS: Metagenomic next- generation sequencing
HIV: Human immunodeficiency virus
AP: Pemetrexed combined with platinum-based
TP: Paclitaxel combined with platinum-based
PCR: Polymerase chain reaction
CRP: C- reactive protein
PCT: Procalcitonin
SAA: Serum amyloid A
GM: Galactomannan
